# Validation of Urinary Charged Metabolite Profiles in Colorectal Cancer Using Capillary Electrophoresis-Mass Spectrometry

**DOI:** 10.3390/metabo12010059

**Published:** 2022-01-10

**Authors:** Toru Sakurai, Kenji Katsumata, Ryutaro Udo, Tomoya Tago, Kenta Kasahara, Junichi Mazaki, Hiroshi Kuwabara, Hideaki Kawakita, Masanobu Enomoto, Tetsuo Ishizaki, Yukako Nemoto, Yoshiaki Osaka, Yuichi Nagakawa, Masahiro Sugimoto, Akihiko Tsuchida

**Affiliations:** 1 Department of Gastrointestinal and Pediatric Surgery, Tokyo Medical University, 6-7-1 Nishi-Shinjuku, Shinjukuku, Tokyo 160-0023, Japan; k-katsu@tokyo-med.ac.jp (K.K.); r-udo@tokyo-med.ac.jp (R.U.); bnr32_nur0129@yahoo.co.jp (T.T.); kasadog327@gmail.com (K.K.); junichim@tokyo-med.ac.jp (J.M.); hiroshi.kuwabara.bob@gmail.com (H.K.); menomoto@yahoo.co.jp (M.E.); wbc15000@yahoo.co.jp (T.I.); naga@tokyo-med.ac.jp (Y.N.); akibobo@hotmail.com (A.T.); 2 Department of Surgery, Kohsei Chuo General Hospital, 1-11-27 Mita, Meguroku, Tokyo 153-0062, Japan; hideakikawakita@gmail.com (H.K.); yosaka913@yahoo.co.jp (Y.O.); 3 Department of Gastroenterology, Kohsei Chuo General Hospital, 1-11-27 Mita, Meguroku, Tokyo 153-0062, Japan; yukako.nemoto@med.tpho-u.ac.jp; 4 Institute for Advanced Biosciences, Keio University, Tsuruoka, Yamagata 997-0811, Japan; mshrsgmt@tokyo-med.ac.jp; 5 Research and Development Center for Minimally Invasive Therapies, Institute of Medical Science, Tokyo Medical University, 6-1-1 Shinjuku, Shinjukuku, Tokyo 160-0022, Japan

**Keywords:** colorectal cancer, adenoma, capillary electrophoresis-mass spectrometry, metabolome

## Abstract

This study aimed to validate and reanalyze urinary biomarkers for detecting colorectal cancers (CRCs). We previously conducted urinary metabolomic analyses using capillary electrophoresis-mass spectrometry and found a significant difference in various metabolites, especially polyamines, between patients with CRC and healthy controls (HC). We analyzed additional samples and confirmed consistency between the newly and previously analyzed data. In total, we included 36 HC, 34 adenoma (AD), and 214 CRC samples, which were used for subsequent analyses. Among the 132 quantified metabolites, 16 exhibited consistent differences in both datasets, which included polyamines, etc. Pathway analyses of the integrated data revealed significant differences in many metabolites, such as glutamine, and metabolites of the TCA (tricarboxylic acid cycle) and urea cycles. The discrimination ability of the combination of multiple metabolites among the three groups was evaluated, which yielded higher sensitivity than tumor markers. The Mann–Whitney test was employed to evaluate the prognosis predictivity of the assessed metabolites and the difference between the patients with or without recurrence, which yielded 16 significantly different metabolites. Among these 16 metabolites, 11 presented significant prognosis predictivity. These data indicated the potential of metabolite-based discrimination of patients with CRC and AD from HC and prognosis predictivity of the monitored metabolites.

## 1. Introduction

Colorectal cancer (CRC) has been reported to have the third-highest cancer-related mortality rate in Japan in 2019 [1]. This may be because the screening rate in Japan is still low compared with that in the United States [2], and by the time patients are diagnosed, cancer progresses to the advanced stage. Most CRC occurs owing to malignant transformation of benign polyps—the so-called adenoma (AD)–carcinoma sequence [3]. AD can be cured by endoscopic resection, and approximately 95% of the early-stage CRC cases can be treated. However, early detection is still difficult owing to the lack of symptoms [4]. Moreover, a large number of patients present recurrence after curative resection in stages II and III of CRC. There are no effective noninvasive approaches that can successfully provide diagnosis, prognosis, survival, and recurrence of CRC in the clinic. Thus, the development of novel screening methods with high sensitivity and specificity at the early stages of CRC and AD and prognosis predictivity is thus essential.

The fecal occult blood test (FOBT) is a screening method commonly used in Japan, and it has contributed to increased chances of early detection of CRC. However, only approximately 3% and 50% of FOBT-positive subjects are diagnosed with CRC and polyps, respectively [5]. The sensitivity of 2-day immunological FOBTs is 60–70% for early-stage and 86% for advanced CRC. Conversely, 36.3% of the CRCs are false negatives, including 25% of advanced CRCs [6]. Therefore, FOBT alone is not a sufficient screening tool.

A colonoscopy is a screening test that allows direct observation of the inside of the colon. The sensitivity of colonoscopy to detect colorectal tumors is as high as 79–100% for CRC and AD with a diameter of ≥10 mm [7]. However, for AD with a diameter of <10 mm, the sensitivity is only 75–85% [8]. Thus, the sensitivity of endoscopy after a positive FOBT is not high despite its invasiveness. Tumor antigens, such as the carcinoembryonic antigen (CEA), a protein marker in the blood, increase the sensitivity and specificity to 18% in stage I, 35% in stage II, and approximately 50% in stage III as a general CRC screening test. However, these sensitivities are not sufficient [9].

Recently, K-RAS has attracted attention as a new genetic marker for CRC [10,11]. It is useful for advanced cancer and for detecting the effects of chemotherapy; however, its effectiveness in early diagnosis remains low. Therefore, the development of a screening tool that is minimally invasive and has higher sensitivity and specificity is necessary.

The recurrence rate after curative resection of colorectal cancer in Japan in 2007 was 15.0% for stage II and 31.8% for stage III [12]. This is clearly a relatively low value, and the prediction of the prognosis before resection and plan treatment is essential. The role of the systemic inflammatory response, as indicated by the neutrophil-to-lymphocyte ratio (NLR), in cancer recurrence and death has been increasingly recognized [13]. The development of a screening tool that is minimally invasive and has higher sensitivity and specificity is thus necessary. Therefore, we analyzed the prognosis predictability using the detected metabolites and compared it with that of NLR.

Recently, not only sputum and blood but also urine is attracting attention as a body fluid that reflects various pieces of information in the body and could be a source of molecular markers. Compared with other body fluids, urine samples contain various metabolites, are not heavily affected by sample collection conditions, including diurnal fluctuations, lifestyle, environmental factors, effects of oral conditions, and sample handling and data analysis methods, and are noninvasive and easy to collect. By identifying metabolites with high sensitivity and specificity to CRC and AD, urine screening tests could replace FOBT in the future. Udo et al. [14] identified and quantified metabolites from urine samples of subjects with CRC at stages 0 to IV or polyps and healthy controls (HC). They found several metabolites in stage IV cancer, identified their metabolic pathways, and demonstrated that further studies are needed for early diagnosis. Therefore, to develop a screening tool that is minimally invasive and has higher sensitivity and specificity, we collected urine samples from subjects with CRC or AD as well as HC. We then conducted a metabolomic analysis to verify our previous results and identify new findings.

## 2. Results

We included 214 patients with CRC (117 men, 97 women) (Table 1), 34 with AD (26 men, 8 women), and 36 with HC (28 men, 8 women).

**Figure 1:**
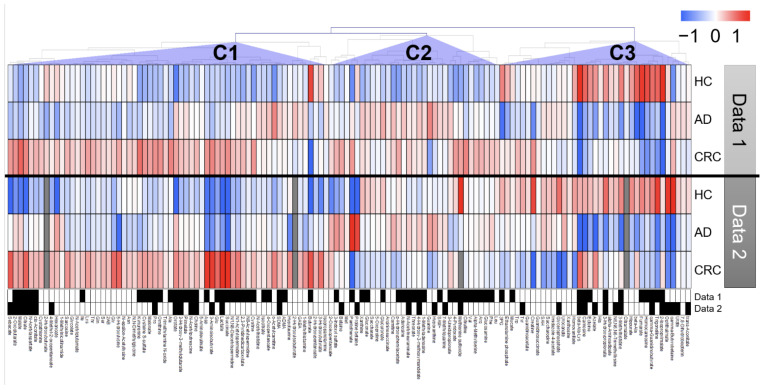
Heatmap of urinary metabolomic profiles. Clustering was performed by combining data 1 and data 2. Fold changes (FC) were calculated by dividing each metabolite concentration by the average of all patients in each dataset. Average FC was calculated for each group, and log_2_ of FC was used to determine the colors that represent higher (red) or lower (blue) concentrations compared with the average (white). Pearson’s correlation was used to cluster metabolites, and prominent clusters are labeled (C1), (C2), and (C3). The metabolites showing *p* < 0.05 (Mann–Whitney U test of healthy controls (HC) vs. adenoma (AD) + colorectal cancer (CRC) cases) are presented in black boxes.

**Figure 2:**
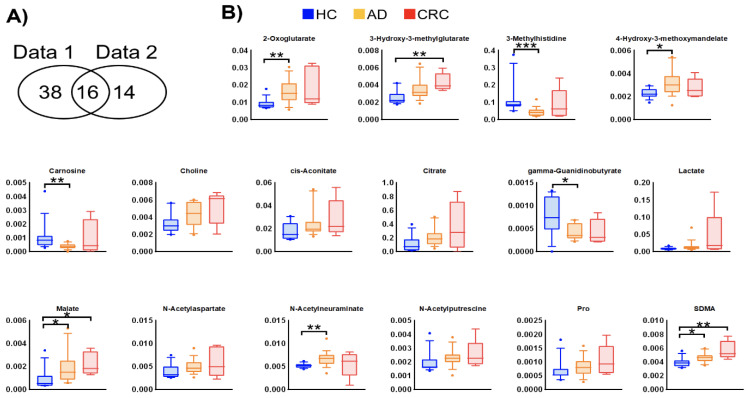
Metabolites showing consistent changes between data 1 and data 2. (**A**) Venn diagram of metabolites with a significant difference (*p* < 0.05, Mann–Whitney U test in HC vs. AD + CRC) in both datasets. (**B**) Box plots of data 2 of each metabolite. The Y-axis is the concentration (µM). Horizontal bars indicate 90%, 75%, 50%, 25%, and 10%, and data on the outside are plotted. * *p* < 0.05, ** *p* < 0.01, and *** *p* < 0.001 (Dunn’s multiple comparison test following the Kruskal–Wallis test).

Among the quantified urinary metabolite concentrations, 132 metabolites were observed in >50% of samples from the previous (data 1) and newly analyzed (data 2) data. The clustered metabolites were visualized as a heatmap (Figure 1). The heatmap shows consistent changes between the two datasets, e.g., the metabolites in cluster C1 and cluster C2 exhibited higher concentrations in CRC and HC, respectively, in both datasets. In the comparison between HC and AD + CRC, 54 and 30 metabolites showed significant differences (*p* < 0.05, Mann–Whitney U test) in data 1 and data 2, respectively, and of these, 16 metabolites were consistently elevated in both data 1 and data 2 (Figure 2). Except for γ-guanidinobutyrate, the concentrations of all the other metabolites were higher in CRC than in HC and AD (Figure 2A and Figure S1).

**Figure 3:**
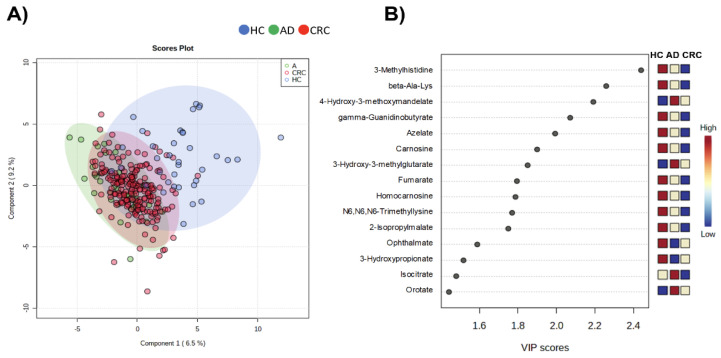
PLS-DA using integrated datasets (data 1 and data 2). (**A**) Score plots and (**B**) VID score. The frequently detected metabolites (≥50% of samples) showing significant differences among HC, AD, and CRC (*p* < 0.05, Kruskal–Wallis test) were used, and the “normalization by sum” and “auto-scaling” options were used. The R2 and Q2 using 10-fold cross-validation were 0.409 and 0.307, respectively, using the two components.

**Figure 4:**
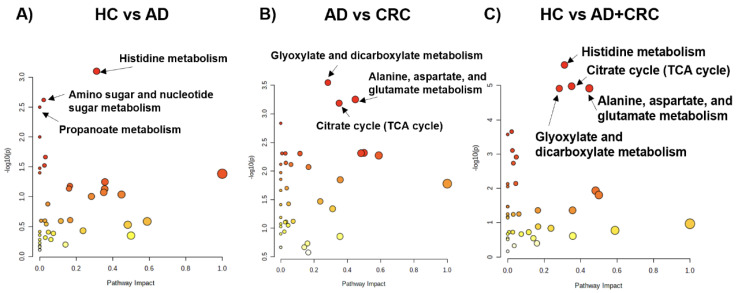
Pathway analysis using integrated datasets (data 1 and data 2). (**A**) HC vs. AD, (**B**) AD vs. CRC, and (**C**) HC vs. AD + CRC. The *x* and *y*-axes indicate pathways that impact and –log_10_P, respectively. The pathways showing (**A**) *p* < 0.05, (**B**) *p* < 0.01, and (**C**) *p* < 0.001 are labeled with pathway names. Red and yellow indicated higher and lower *P*-values. Larger dots indicate a higher impact score.

**Figure 5:**
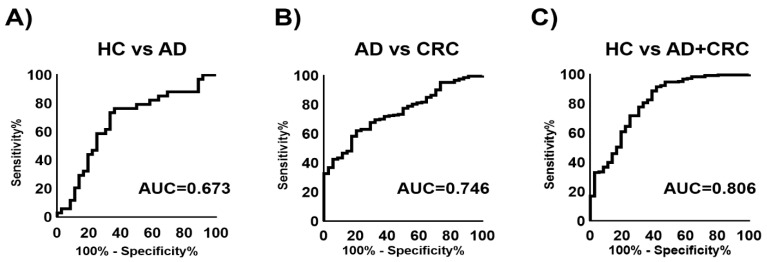
Receiver operating characteristic (ROC) curves of multiple logistic regression (MLR) models using integrated datasets (data 1 and data 2). (**A**) HC vs. AD, (**B**) AD vs. CRC, and (**C**) HC vs. AD + CRC. The metabolites and parameters of the MLR models are listed in Table 2, Table 3 and Table 4.

To evaluate the discrimination ability of the overall metabolomic profile, partial least-squares discriminant analysis (PLS-DA) was conducted (Figure 3). The HC groups were separated from the other groups; however, AD and CRC exhibited considerable overlap, indicating that the overall metabolomic profile of HC had the largest distinction from the other two groups. We also evaluated pathway-level differences in the metabolomic profiles between the two groups (Figure 4). Comparison between HC and AD (Figure 4A), AD and CRC (Figure 4B), and HC and AD + CRC (Figure 4C) revealed more significantly different pathways. Histidine metabolites were significantly different when comparison was made between HC and AD (Figure 4A) as well as between HC and AD + CRC (Figure 4C). Two pathways, namely (1) alanine, aspartate, and glutamate metabolism and (2) the citrate cycle (TCA cycle), are connected, and the metabolite concentrations of the TCA cycle, urea cycle, glutamine pathway, histidine metabolism, and polyamine pathways are depicted in the comparison between HC and AD + CRC (Figure 5). The other comparisons are presented in Figures S2 and S3.

**Figure 6:**
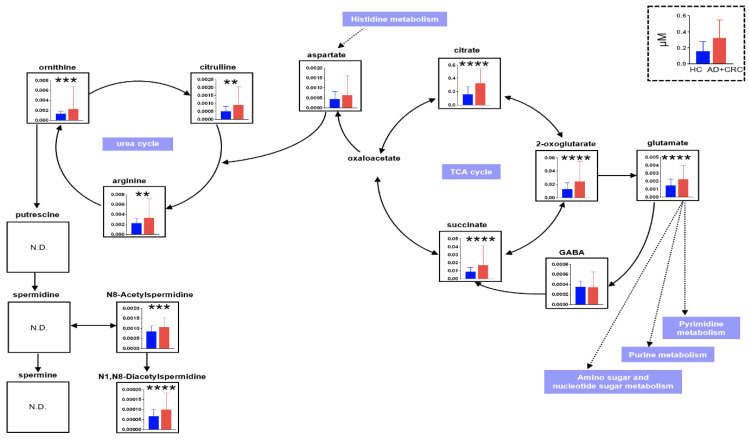
Comparison of metabolite concentrations between HC vs. AD + CRC in the TCA cycle, glutamine pathway, urea cycle, and polyamine pathways. The datasets include both data 1 and data 2. ** *p* < 0.01, *** *p* < 0.001, and **** *p* < 0.0001 (Mann–Whitney U test). ”ND” is “No Data”.

The ability of the combination of multiple metabolites to discriminate between affected cases and HC was also evaluated. Multiple logistic regression (MLR) models were developed to discriminate AD from HC (AUC = 0.673), CRC from AD (AUC = 0.746), and AD + CRC from HC (AUC = 0.806) (Figure 6). The metabolites used for these models are listed in Table 2, Table 3 and Table 4. Using the MLR model to discriminate AD + CRC from HC, the sensitivity was compared with those of CEA and CA19-9 (Table 5). The sensitivities of CEA, CA19-9, and MLR were 69.0%, 82.2%, and 85.2%, respectively. When comparing HC and AD + CRC, *N*^1^,*N*^8^-diacetylspermidine, 2-oxoglutarate, and citrate showed significant differences (*p* < 0.05, Mann–Whitney U test) (Tables S1–S4).

**Figure 7:**
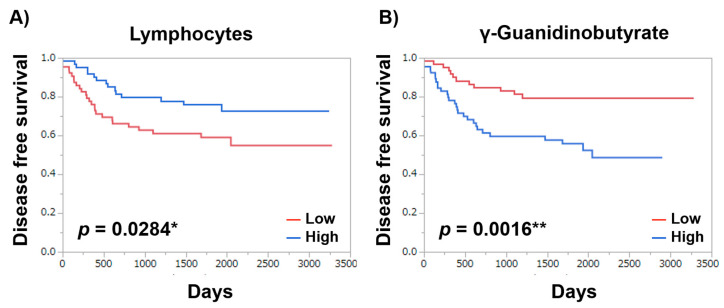
Kaplan–Meier curves of disease-free survival. (**A**) Lymphocytes and (**B**) γ-Guanidinobutyrate. NLR ≥ 3 and median value of γ-Guanidinobutyrate were used as thresholds to categorize the patients into high and low groups. *p*-value was calculated using the Log-rank test. *p* < 0.05 *, *p* < 0.01 **.

To evaluate the prognosis predictivity of the profiled metabolites, the Mann–Whitney test was used for evaluating the difference between the patients with or without recurrence, which resulted in 16 significantly different metabolites (*p* < 0.05). Among these metabolites, 11 metabolites presented significantly high prognosis predictivity (*p* < 0.05; rank-log test) (Table 6). γ-guanidinobutyrate presented the highest predictive ability (*p*-value = 0.0016) in these patients. Asn, 3-Methylhistidine, 1-Metyladenosine, 3-Hydroxybutyrate, N-Acetylglutamate, Hippurate, Met, 7,8-Dihydrobiopterin, and Sebacate also presented significantly high prognosis predictivity (*p* < 0.05; rank-log test). Kaplan–Meier curves of disease-free survival demonstrated that disease-free survival increases when the -Guanidinobutyrate level is lower (*p* = 0.0016) or lymphocyte count is higher (*p*-value = 0.0284) (Figure 7).

## 3. Discussion

In this study, citrate, 2-oxoglutarate, and succinate in the TCA cycle; glutamate leading to the pyrimidine pathway and the purine pathway; amino sugar and nucleotide sugar metabolism; and citrulline, arginine, and ornithine in the urea cycle leading to spermidine were significantly identified in the HC group compared with the AD + CRC group. N^8^-Acetylspermidine and *N*^1^,*N*^8^-diacetylspermidine metabolized from spermidine were also significantly detected. Thus, in CRC, there may be changes in the TCA cycle and energy production using the glutamate-mediated pyrimidine as well as the nucleic acid synthesis and urea cycle-mediated spermidine pathways. The only substance that was significantly different between the AD and HC groups was 2-oxoglutarate, which was significantly elevated in patients with cancer; thus, no metabolites specific to AD were identified. In MLR (HC vs. AD + CRC), which included a combination of N^8^-acetylspermidine, *N*^1^,*N*^8^-diacetylspermidine, and 2-oxoglutarate, the positive rate was higher and the negative rate was lower than that of CEA and CA19-9 (Table 5). To distinguish the AD + CRC group from the HC group as well as the CRC group from the AD group, combining several metabolites instead of using a single substance as a marker may yield better results. Metabolomics comprehensively measures small molecules called metabolites, allows the analysis of cellular functions that change these metabolites owing to several factors such as the environment and diseases, and enables the development of diagnostic applications for diseases. Nuclear magnetic resonance (NMR) [15] and mass spectrometry (MS) [16] are the main measurement instruments in this field. However, because NMR has low sensitivity and the number of substances that can be measured simultaneously is limited, the use of MS for comprehensive measurement is common. Because it is not possible to individually quantify substances with the same *m*/*z* when MS is used alone, a combined use of MS and separation techniques, such as gas chromatography, liquid chromatography, and capillary electrophoresis, is common [17,18,19]. Each method is best at analyzing different types of targets, and it is not possible to detect all metabolite measurements using a single method.

Therefore, the appropriate selection is required according to the targeted metabolites. Soga et al. used capillary electrophoresis–time-of-flight MS (CE–TOFMS) to measure comprehensive metabolic profiles in colorectal and gastric cancer tissues [20]. CE–TOFMS specializes in the measurement of ionic substances, which represent the majority of the major metabolites involved in energy metabolisms, such as glycolysis, pentose phosphate pathway, central carbon metabolism, and nucleic acid synthesis represented by the TCA cycle, and metabolites involved in amino acid biosynthesis/degradation. Therefore, it is the best method for studying cancers in which metabolic disorders involving these pathways are common and for identifying biomarkers at the metabolic level. Thus, this method was also used in this study.

Metabolic profiles are easier to determine than transcriptomes and proteomes and are more quantitative and reproducible, so they have great advantages when considering clinical applications. However, because metabolic profiles are susceptible to fluctuations due to environmental factors, searching for marker candidates by minimizing the effects of factors other than diseases or by analyzing metabolites while considering factors such as lifestyle is important. Therefore, in this study, we studied markers and metabolic pathways for AD/CRC by conducting metabolomic analysis using urine, which is less susceptible to confounding effects and can be collected in a minimally invasive manner. Compared with other body fluids, saliva and urine have major advantages, such as low invasiveness of collection, low cost, and safe sample collection. However, the reproducibility is low unless sample collection conditions, including diurnal fluctuations, lifestyle, environmental factors, effects of oral conditions, sample handling, and data analysis methods, are standardized and well established. Moreover, urine metabolites can be inaccurate in cases of renal dysfunction, dehydration, and urinary tract infections. However, a previous study reported that urinary metabolites were more stable than saliva metabolites and were not affected by diurnal variation. Thus, we used urinary metabolites for this metabolomic analysis.

The Warburg effect has been reported in cancer cells, in which ATP is produced more through glycolysis than through oxidative phosphorylation of mitochondria in aerobic and anaerobic environments [21,22]. Hypoxia-inducible factor-1 (HIF-1) is activated when tumor cells become hypoxic as the tumor grows. HIF-1 promotes lactate production by enhancing the expression of pyruvate kinase. It also suppresses pyruvate dehydrogenase, inhibits the production of acetyl-CoA from pyruvate, and reduces energy production in the mitochondria [23]. Thus, energy may be produced using a pathway other than oxidative phosphorylation, such as through glycolysis, which has very low ATP production efficiency compared with oxidative phosphorylation but has a high ATP production rate and does not require oxygen. Therefore, it is an effective method for energy production in the hypoxic environment of malignant tumors. Furthermore, under hyperglycemic conditions, the pentose phosphate pathway, which is parallel to glycolysis, is enhanced, and nucleic acid/protein/fatty acid synthesis is increased to produce energy [24]. In malignant tumors, energy is produced using glycolysis and nucleic acid/protein/fatty acid synthesis in addition to the TCA cycle.

The APC gene, a Myc suppressor gene, has been reported to be mutated in 20–50% of patients with cancer and in approximately 30% of AD in non-polyposis patients [3,25]. Moreover, a significant upregulation of the c-Myc protein is observed in 70% of CRC [26,27,28]. Thus, degeneration of the c-Myc protein due to *APC* mutations in the progression from AD to cancer in the AD–carcinoma sequence is supported by the metabolomic findings of this study.

Soga et al. demonstrated that (1) CRC metabolism changes from the benign tumor stage but does not depend on the stage; (2) the Myc protein, an oncogene product, alters the metabolism of CRC through 215 metabolic reactions; (3) suppression of Myc and metabolic enzymes controlled by Myc inhibits the growth of CRC cells; and (4) Myc-controlled pyrimidine metabolic pathways are promising targets for CRC treatment [29]. In the AD–carcinoma sequence, AD and cancer have the same metabolic pathway, and there is a possibility that metabolism is promoted by the Myc expression. We previously reported that the results were similar from early-stage cancer to advanced cancer without distant metastasis and that the expression level and type of metabolites were completely different in advanced cancer with distant metastasis [14]. Thus, it was inferred that completely different metabolites are expressed from primary cancer or distant metastases. This may also be valid in cases of postoperative recurrence and distant metastases.

The intracellular nucleic acid synthesis produces energy by synthesizing nucleotides from the phosphorylation of pyrimidine and purine bases, which is important not only for screening but also for clinical considerations. Degraded nucleic acids are recycled into nucleotides again through the salvage synthesis system. 5-fluorouracil inhibits the salvage synthesis system in CRC. Our study provides indirect evidence for this mechanism. Energy is produced during this stage of the benign tumor using various metabolic pathways in CRC. Thus, the identification of metabolites involved in energy metabolism pathways may be useful for screening markers for AD and cancer, elucidating pathological conditions, and developing therapeutic methods for metabolic inhibition.

In this study, urinary metabolites in the CRC, AD, and HC groups were identified. These polyp lesions have different carcinogenic processes, and the described subtypes can be distinguished using the metabolomic approach presented in this study. We discovered that cancers and polyps potentially use the same metabolic pathways, and it is possible to distinguish among HC, AD, and CRC by combining some of the metabolites identified in this study. Furthermore, the sensitivity was not inferior to that of tumor markers. γ-Guanidinobutyrate is an amino acid in the urea cycle and presented the highest predictive ability. It was discovered that it could be possible to detect molecules with a higher prognosis than NLR. It was revealed that metabolomic analysis of colorectal cancer might enable a more accurate prognosis prediction. However, a single marker that distinguishes the HC group from the AD and CRC groups has not been identified. Moreover, finding specific metabolites is difficult owing to the stage of AD–cancer development. When CRC progression was distinguished by stages in this study, there was no significant difference between HC and AD subjects. Comparison among HC, AD, and CRC without distinguishing the AD subtypes (tubular, villous, and serrated) is the limitation of this study. In conclusion, metabolomic analysis is a developing field and is expected to aid in the advancement of screening of markers and prognosis prediction for AD and cancer, elucidation of the mechanisms underlying pathological conditions, and development of novel therapeutic agents.

## 4. Materials and Methods

### 4.1. Study Subjects

In this study, we collected urinary samples from patients with CRC (*n* = 5), AD (*n* = 18), and HC (*n* = 14). The characteristics of the subjects are summarized in Table 1. This study was conducted according to the guidelines of the Declaration of Helsinki. The study protocol was approved by the Ethics Committee at Tokyo Medical University (study approval #2346). Written informed consent was obtained from each subject before study participation.

### 4.2. Metabolomic Analysis and Data Analysis

We conducted a CE–TOFMS-based metabolomic analysis of the urine samples. The protocols for sample collection, processing, and measurement instruments with their parameters were as per a previous study [14]. Briefly, the 154 metabolites observed in the previous study [14] were analyzed. The absolute concentration divided by the creatinine concentration of each sample was calculated.

The differences among the CRC, AD, and HC groups were analyzed using the Kruskal–Wallis test together with Dunn’s multiple comparison test. For two-group comparisons, e.g., HC vs. CRC + AD, the Mann–Whitney U test was employed. To evaluate the overall metabolic profile clustering, PLS-DA and pathway analyses were conducted. The ability to discriminate between the case groups and controls was assessed using the area under the receiver operating characteristic (ROC) curves (AUC). To evaluate the discrimination abilities of combinations of multiple metabolites, MLR models were developed. Two polyamines, namely *N*^1^,*N*^8^-diacetylspermidine and N8-acetylspermidine, which were reported as markers in our previous study [30], were used as independent variables. Forward stepwise feature selection (criterion: *p* = 0.05) was used to select additional metabolites as independent variables. The optimal cutoff values of the MLR models were determined using the ROC curves. The sensitivities of the MLR model and tumor markers, including serum CEA and CA19-9, were compared.

Among clinical parameters, NLR, WBC, and neutrophils did not show any significant predictivity, whereas lymphocytes showed significant predictivity (*p* = 0.00284). As a prognostic factor, relapse-free survival was analyzed using the CRC data. To assess prognosis-relevant metabolites, the Mann–Whitney test was used to determine the candidate metabolites with high prognosis predictivity. Based on the median value of each candidate metabolite, the data were classified into higher and lower risk groups. The prognosis predictivity of disease-free survival (DFS) was evaluated using the Kaplan–Meier curve and log-rank test. Data for recurrence, relapse, and metastasis were collected. Other clinical parameters, NLR, WBC, neutrophils, and lymphocytes, were also evaluated. High and low groups were divided based on NLR of ≥3 and median values of other parameters. Patients with missing values were eliminated depending on the parameters to be evaluated, and the patients at the median value were grouped as a high group when the patient number was odd.

For data analyses, MeV TM4 (ver. 4.9.0, http://mev.tm4.org/, accessed on 6 September 2021), GraphPad Prism (v.9.2.0, GraphPad Software Inc., San Diego, CA, USA), JMP Pro (v. 14.1.0, SAS Institute Inc., Cary, NC, USA), and MetaboAnalyst (v. 5.0, https://www.metaboanalyst.ca/, accessed on 6 September 2021) were used.

## Figures and Tables

**Table 1:** Subjects’ characteristics.

Group		Age		Gender	
	*n*	Mean	SD	Male	Female
HC	36	49.1	12.2	28	8
AD	34	65.9	14.0	26	8
CRC	214	68.8	11.8	117	97

**Table 2:** MLR model to discriminate AD from HC.

HC vs. AD	Parameter	95% CI		Odds Ratio	95% CI		*p*
*N*^8^-Acetylspermidine	3.94 × 10^3^	−1.53 × 10^3^	2.32 × 10^3^	2.03	0.0650	63.2	0.69
*N*^1^,*N*^8^-Diacetylspermidine	3.94 × 10^3^	−9.57 × 10^3^	1.74 × 10^4^	2.68	0.0910	79.2	0.57
2-Oxoglutarate	29.1	−29.9	88.0	3.51	0.275	44.7	0.33
(Intercept)	−1.10	−2.59	0.401	-	-	-	0.15

**Table 3:** MLR model to discriminate CRC from AD.

AD vs. CRC	Parameter	95% CI		Odds Ratio	95% CI		*p*
*N*^8^-Acetylspermidine	1.24 × 10^2^	−1.09 × 10^3^	1.45 × 10^3^	1.67	0.0110	4.12 × 10^2^	0.85
*N*^1^,*N*^8^-Diacetylspermidine	5.01 × 10^3^	−4006.923	1.43 × 10^4^	1.25 × 10^2^	0.0210	9.98 × 10^5^	0.28
Citrate	5.19	2.30	8.59	6.18 × 10^3^	47.9	1.87 × 10^6^	0.0010
Citrulline	1.11 × 10^3^	2.37 × 10^2^	2.22 × 10^4^	2.04 × 10^5^	13.6	4.16 × 10^10^	0.030
(Intercept)	−0.780	−2.26	0.575	-	-	-	0.28

**Table 4:** MLR model to discriminate CRC + AD from HC.

HC vs. AD + CRC	Parameter	95% CI		Odds Ratio	95% CI		*p*
*N*^8^-Acetylspermidine	1.27 × 10^3^	−1.49 × 10^2^	2.81 × 10^3^	1.94 × 10^2^	0.540	1.15 × 10^5^	0.090
*N*^1^,*N*^8^-Diacetylspermidine	8.20 × 10^3^	−1.86 × 10^3^	1.87 × 10^4^	2.73 × 10^3^	0.167	6.85 × 10^7^	0.12
Citrate	7.88	4.56	11.7	5.69 × 10^5^	2135.038	3.68 × 10^8^	<0.0001
(Intercept)	−1.58	−3.11	−0.183	-	-	-	0.030

CI: confidence interval; GC: gastric cancer.

**Table 5:** Comparison of the positive rate between tumor markers and multiple logistic regression (MLR) model.

	Positive		Negative		Total
	(*n*)	(%)	(*n*)	(%)	(*n*)
CEA	147	69	66	31	213
CA19-9	175	82.2	38	17.8	213
MLR (HC vs. AD + CRC)	242	85.2	42	14.8	284

**Table 6:** *p*-values of DFS predictive factors.

Item	Log-Rank Test	Wilcoxon Test
Metabolites		
γ-Guanidinobutyrate	0.0016 **	0.0028 **
Asn	0.0315 *	0.0400 *
3-Methylhistidine	0.0365 *	0.0731
1-Metyladenosine	0.0348 *	0.0404 *
3-Hydroxybutyrate	0.0297 *	0.0390 *
N-Acetylglutamate	0.0046 **	0.0132 *
Hippurate	0.0162 *	0.0277 *
Met	0.0437 *	0.0599
7,8-Dihydrobiopterin	0.0434 *	0.0831
GABA	0.0525 **	0.0235 *
Sebacate	0.0063 **	0.0025 **
Other clinical parameters		
NLR	0.1183	0.1369
WBC	0.2164	0.2886
Neutrophils	0.0754	0.0585
Lymphocytes	0.0284 *	0.0193 *

*p*-Value was calculated using the Log-rank test. *p* < 0.05 *, *p* < 0.01 **.

## Data Availability

The data presented in this study are available on request from the corresponding author. The data are not publicly available due to prevent misuse.

## Supplementary Materials

The following are available online at https://www.mdpi.com/article/10.3390/metabo12010059/s1, Figure S1. Metabolites showing consistent changes between data 1 and data 2. Box plots of data 1 of each metabolite, Figure S2. Comparison of metabolite concentrations between HC vs. AD, Figure S3. Comparison of metabolite concentrations between HC vs. AD + CRC, Table S1. AUC value of *N*^8^-acetylspermidine, Table S2. AUC value of *N*^1^,*N*^8^-diacetylspermidine, Table S3. AUC value of 2-oxoglutarate, Table S4. AUC value of citrate.
