# Supplementary material for: Validation of Urinary Charged Metabolite Profiles in Colorectal Cancer Using Capillary Electrophoresis-Mass Spectrometry
Source: Metabolites. 2022 Jan 10;12(1):59. doi: 10.3390/metabo12010059 (PMC8779129; doi:10.3390/metabo12010059)
Supplement: Supplementary file 1 [file metabolites-12-00059-s001.zip › metabolites-1486764-supplementary.pdf]

metabolites

MDPI

Supplementary Material

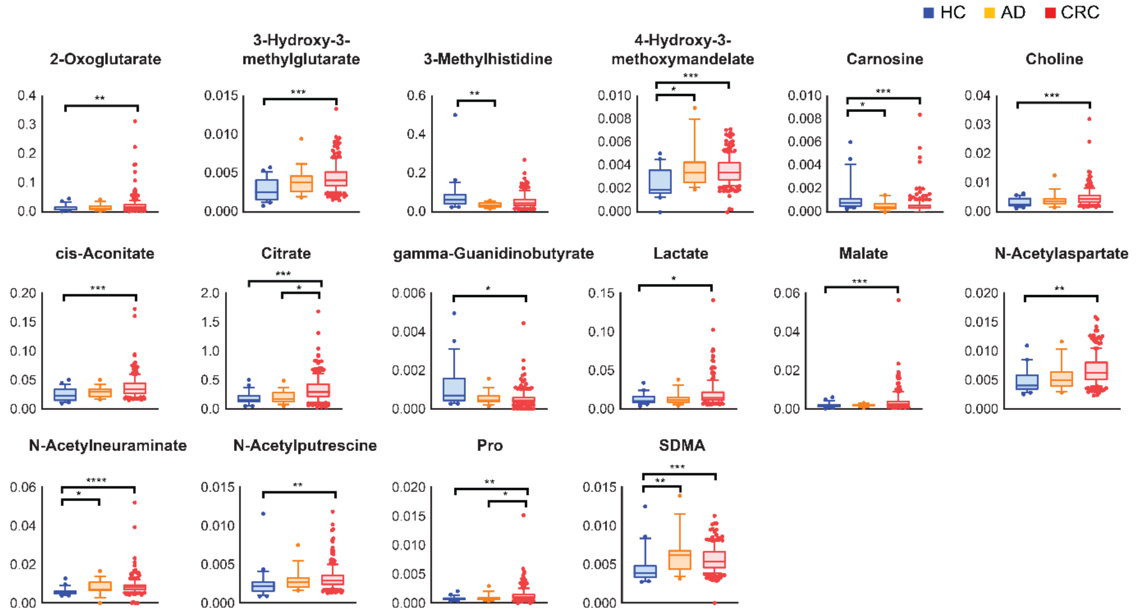

Figure S1. Metabolites showing consistent changes between data 1 and data 2. Box plots of data 1 of each metabolite. See the legend of Figure 2 for details.

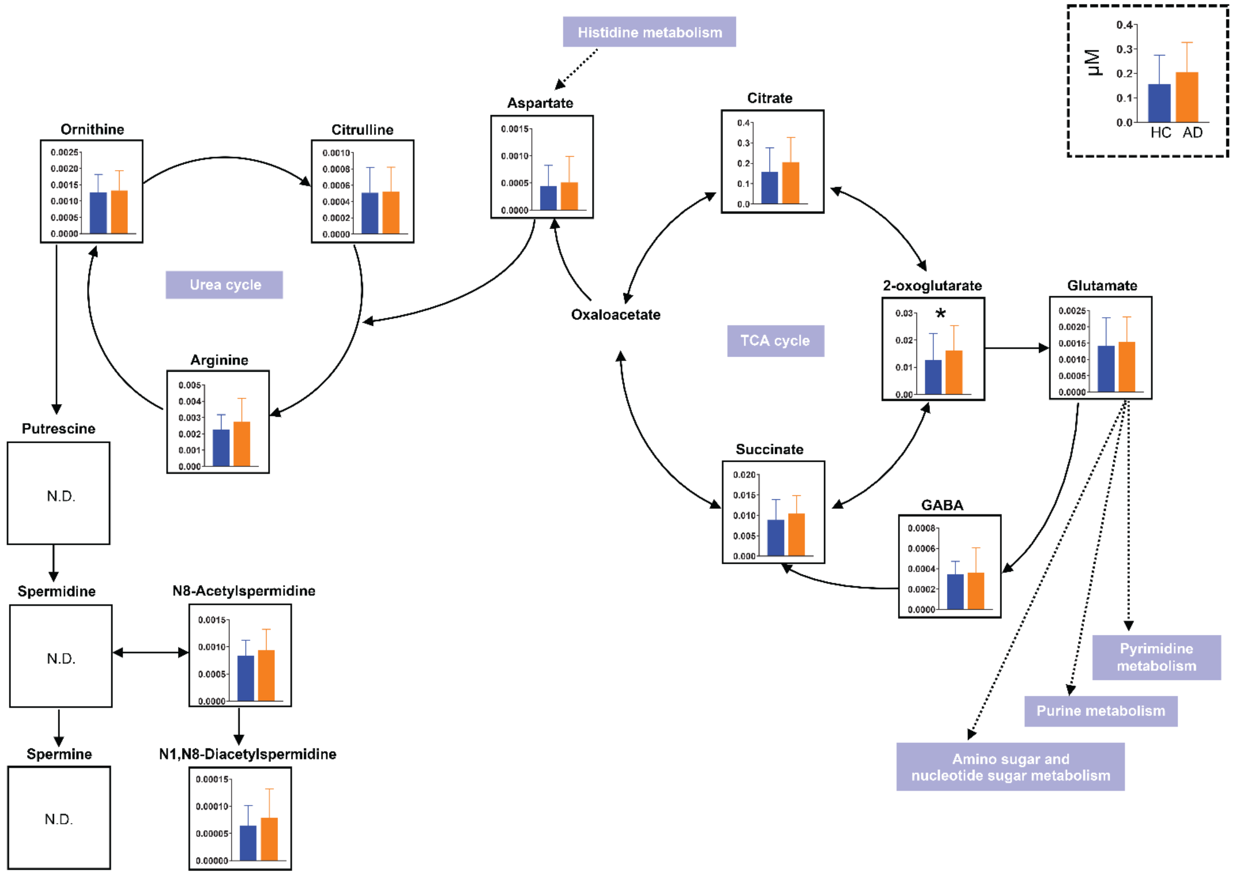

Figure S2. Comparison of metabolite concentrations between HC vs. AD. See the legend of Figure 6 for details.

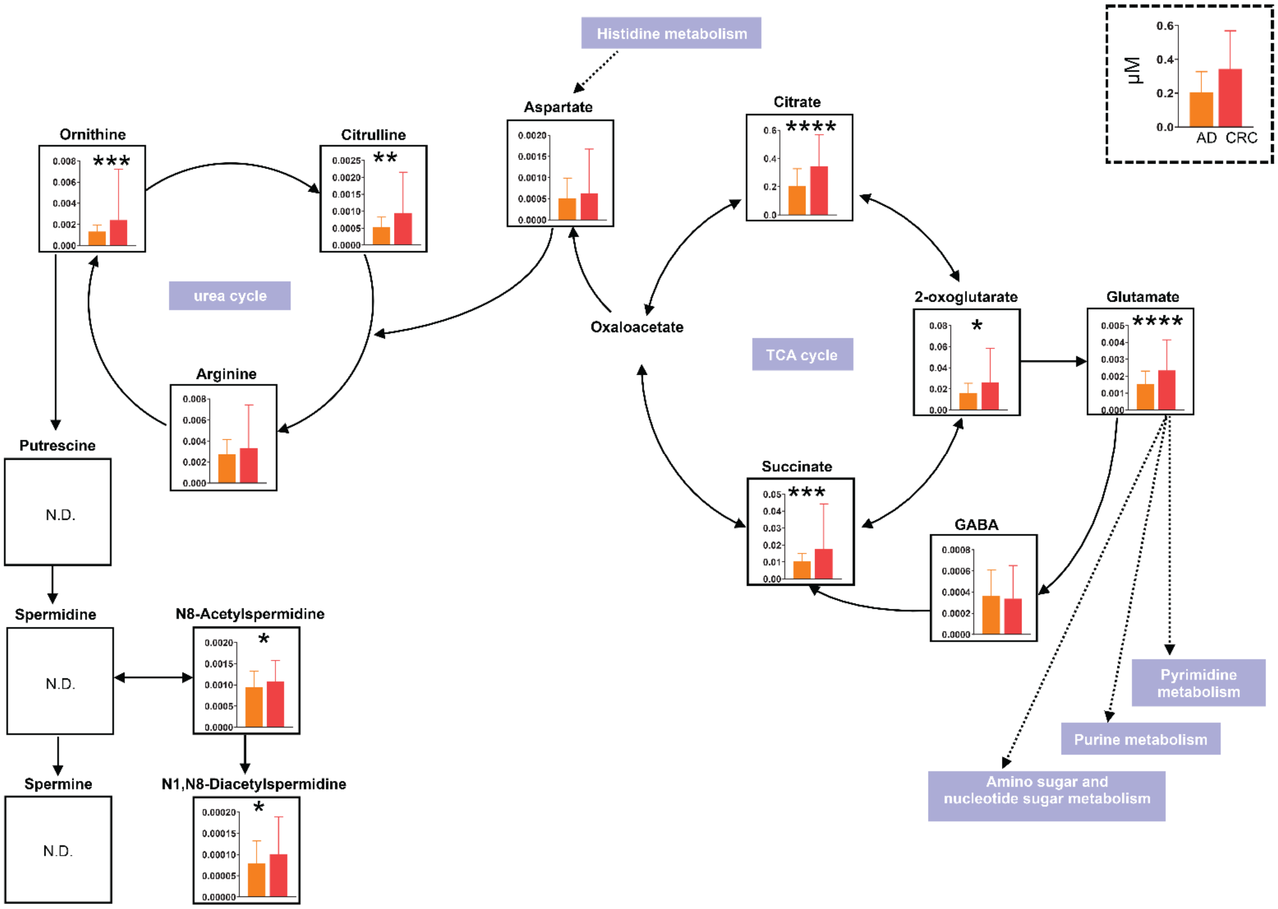

Figure S3. Comparison of metabolite concentrations between HC vs. AD + CRC. See the legend of Figure 6 for details.

Table S1. AUC value of N^8-acetylspermidine.

N^8-Acetylspermidine	AUC	95% CI		*P*
HC vs. AD	0.581	0.443	0.719	0.24
AD vs. CRC	0.609	0.510	0.707	0.04
HC vs. AD + CRC	0.682	0.600	0.763	0.00

AUC: area under the curve, CI: confidence interval, HC: healthy controls, AD: adenoma, CRC: colorectal cancer.

Table S2. AUC value of N^1,N^8-diacetylspermidine.

N^1,N^8-Diacetylspermidine	AUC	95% CI		*P*
HC vs. AD	0.626	0.492	0.760	0.07
AD vs. CRC	0.623	0.532	0.715	0.02
HC vs. AD + CRC	0.706	0.626	0.785	<0.0001

AUC: area under the curve, CI: confidence interval, HC: healthy controls, AD: adenoma, CRC: colorectal cancer.

Table S3. AUC value of 2-oxoglutarate.

2-Oxoglutarate	AUC	95% CI		*P*
HC vs. AD	0.666	0.536	0.796	0.66
AD vs. CRC	0.631	0.534	0.729	0.84
HC vs. AD + CRC	0.748	0.600	0.763	<0.0001

AUC: area under the curve, CI: confidence interval, HC: healthy controls, AD: adenoma, CRC: colorectal cancer.

Table S4. AUC value of citrate.

Citrate	AUC	95% CI		*P*
HC vs. AD	0.627	0.496	0.757	0.07
AD vs. CRC	0.710	0.622	0.798	<0.0001
HC vs. AD + CRC	0.771	0.692	0.850	<0.0001

AUC: area under the curve, CI: confidence interval, HC: healthy controls, AD: adenoma, CRC: colorectal cancer.
